# Assessment of Possibility of Using Ultrasound Imaging in Treatment of Stress Urinary Incontinence in Women—Preliminary Study

**DOI:** 10.3390/bioengineering12060633

**Published:** 2025-06-10

**Authors:** Gabriela Kołodyńska, Maciej Zalewski, Aleksandra Piątek, Anna Mucha, Krystyna Rożek-Piechura, Waldemar Andrzejewski

**Affiliations:** 1 Department of Physiotherapy, Wroclaw University of Health and Sport Sciences, 51-612 Wrocław, Poland; krystyna.rozek-piechura@awf.wroc.pl (K.R.-P.); waldemar.andrzejewski@awf.wroc.pl (W.A.); 2 Faculty of Medical Sciences and Health Sciences, University of Social and Medical Sciences in Warsaw, 04-367 Warszawa, Poland; 3 Independent Public Health Care Center of the Ministry of the Interior and Administration in Wrocław, Department of Gynaecology, 50-233 Wrocław, Poland; zalewskim@interia.pl (M.Z.); ola.piatek@yahoo.pl (A.P.); 4 Department of Genetics, Wrocław University of Environmental and Life Sciences, 50-375 Wrocław, Poland; anna.mucha@upwr.edu.pl

**Keywords:** electrostimulation, sonofeedback, stress urinary incontinence

## Abstract

The number of people suffering from urinary incontinence increases every year. Along this trend, the knowledge of society increases regarding the various methods available for treating this ailment. Both patients and researchers are constantly looking for new treatments for urinary incontinence. One of the new solutions is sonofeedback of the pelvic floor muscles, which may help to strengthen them and thus reduce the problem. The aim of this study was to evaluate the effectiveness of sonofeedback and transvaginal electrostimulation in increasing the bioelectrical activity of pelvic floor muscles in postmenopausal women with stress urinary incontinence. Sixty women with stress urinary incontinence were enrolled in the study. The patients were divided into two groups: A, where sonofeedback was used, and B, where electrostimulation of the pelvic floor muscles was performed with biofeedback training. In patients, the resting bioelectrical activity of the pelvic floor muscles was assessed using an electromyograph. The assessment of the resting bioelectrical activity of the pelvic floor muscles was performed before the therapy, after the 5th training, and after the therapy. It was observed that after the end of the therapy, the average bioelectrical potential increased by 1.1 µV compared with the baseline in group A. It can be suggested that the sonofeedback method is comparatively effective in reducing symptoms that are associated with urinary incontinence as an electrostimulation method with biofeedback training.

## 1. Introduction

Urinary incontinence is an extremely common problem affecting postmenopausal women. This condition adversely affects numerous aspects of life [1,2]. Urinary incontinence (UI) is characterized as the involuntary leakage of urine due to dysfunction of the urethral sphincters which control the outflow of urine [3]. According to the International Continence Society (ICS), it is also the cause of hygienic difficulties, leading to social problems. UI mainly concerns older women, meaning 10–20% of all women. The latest data show that this problem occurs in as many as 77% of women living in nursing homes [4]. These data show that UI should be considered a social disease, as it affects more than 5% of the population, regardless of ethnic or cultural differences [5].

The incidence of urinary incontinence increases with age. In addition, chronic diseases and low economic status predispose people to incontinence. Numerous researchers state that this problem is most commonly seen in menopausal and postmenopausal women [6,7,8].

One of the important risk factors predisposing the occurrence of UI is menopause. It is caused by a significant decrease in estrogen in the blood, which contributes to the lowering and shortening of the urethra. As a result, it can cause frequent urination. In addition, in menopausal women, the detrusor muscles and those responsible for urine excretion are also very often too weak [9].

Urinary incontinence can also be caused by part of the aging process [10]. Changes in the bladder, pelvic structure, and comorbidities that can interfere with continence mechanisms are major contributors to the onset of incontinence and often occur in older adults. The comorbidities include above all diabetes, disability, or cognitive impairment. Numerous studies have been published showing the relationship between age and incontinence [11].

Different grades of urinary continence can be listed based on the severity of symptoms:

Grade I: urine loss during coughing, sneezing, pressure, and laughing;
Grade II: urine loss during lifting, running, and climbing stairs;
Grade III: urine loss while lying/standing [12].

Conservative treatment is recommended in the presence of I or II grade urinary incontinence. Surgical treatment should only be considered in the case of advanced disease or the presence of disturbances in pelvic floor stability [13,14]. Modern physiotherapeutic methods can reduce the symptoms of urinary incontinence and prevent the occurrence of adverse effects, such as psychological, social, and societal disorders [15]. Physiotherapy-based treatments can often constitute an alternative to surgical treatment or pharmacotherapy [16,17]. Conservative treatment methods primarily include physiotherapy [18]. Properly selected physiotherapy methods ensure improvement in or the complete resolution of symptoms. In the case of incomplete symptom reduction, physiotherapy increases the chance of successful applied surgical treatment [19,20]. Conservative treatment includes physiotherapy (pelvic floor muscle exercises, biological feedback—biofeedback, sonofeedback, pelvic floor muscle electrostimulation, bladder training, magnetic field stimulation); mechanical measures (pessaries, cones, intravaginal balls—Kegel balls); lifestyle changes; and pharmacotherapy [21].

Currently, the common conservative treatment is the electrostimulation of the pelvic floor muscles (PFMs). This method is primarily used for patients who are unable to contract these muscles on their own. The cause of pelvic floor muscle weakness is most often due to impaired conduction of nerve impulses through the pudendal nerve. The purpose of using electrical stimulation is to restore the ability of muscles exhibiting partial denervation to contract [22,23]. The electrical impulses sent by the electrostimulation device cause the muscles to contract, which results in their passive reinforcement. The procedure can be performed as direct stimulation using a special vaginal electrode, as indirect stimulation using surface electrodes, or as functional electrostimulation using both types of electrode simultaneously [24,25].

Another method that can be used in the treatment of stress urinary incontinence is sonofeedback. This method uses biological feedback and, as shown in the studies presented in the available literature, can be used to strengthen the pelvic floor muscles [26,27,28].

Sonofeedback allows you to observe muscle activity in real time. This enables the therapist to teach the patient how to tense and relax the muscles correctly [29]. Sonofeedback is gaining increasing interest [30] and is used by physiotherapists to treat a variety of conditions. It is also referred to as a rehabilitative form of ultrasound imaging. With this innovative method, pelvic floor muscle reinforcement is achieved in patients with urinary incontinence. Furthermore, ultrasound allows the physical therapist to evaluate some of the functions in these muscles.

Ultrasound examination also allows the physiotherapist to assess the function of the pelvic floor muscle. Thanks to the examination, the physiotherapist can assess, among other targets, the urethra, bladder neck, or the pubic symphysis [31]. The methodology is standardized and must be the same for all measurements [32,33].

The aim of study was to evaluate the effectiveness of sonofeedback and transvaginal electrostimulation in increasing the bioelectrical activity of pelvic floor muscles in women.

The research questions of this study are as follows:

- Will the use of sonofeedback training increase the bioelectric activity of the PFMs in the study group?
- Is training with the use of the sonofeedback method as effective in the treatment of SUI as electrostimulation with biofeedback training?

## 2. Material and Methods

This project was carried out at the Gynecology Department of a Independent Public Health Care Center of the Ministry of the Interior and Administration in Wrocław in Wrocław, Poland. The research was approved by the Bioethics Committee of the Medical University in Wroclaw and took place between February and June 2019.

Overall, 60 women diagnosed with stress urinary incontinence qualified for the project. Before therapy, each patient underwent a medical history and physical examination. To participate in the study, the patients had to meet strictly defined inclusion and exclusion criteria.

The patients were randomly assigned to three groups. Simple randomization was used. Sonofeedback training was performed in group A. In group B, the electrostimulation of the pelvic floor muscles was performed, combined with biofeedback training. Group C was the control group, which did not receive the therapy. Patients were recommended not to exercise the PFMs between sessions.

In all subjects, the bioelectrical activity of the pelvic floor muscles was tested three times: before, after the 5th, and after the therapy. Measurement was performed with a MyoPlus 4 Pro Electromyograph. Resting EMG levels were examined for 60 s. Changes in the mean resting bioelectrical activity of the PFMs were evaluated. During the examination, the patients were in the supine position on a stable therapy table. The electrodes were placed in the same way in each subject: there was an endovaginal electrode in the vagina.

Different management strategies were applied to the patients, depending on enrollment to a specific group. In patients from group A, the sonofeedback training of the pelvic floor muscles was performed. After enrollment in the group, patients were trained to perform sonofeedback training correctly. The ability to perform the training correctly was a requirement for participation. The correctness of the training was evidenced by the displacement of the bladder, which both the patient and the therapist could observe on the ultrasound screen. The training was conducted in accordance with the guidelines in the literature [34].

There were two images on the monitor screen during the sonofeedback training. One image showed the lesser pelvis in the resting phase, while the other image was a real image in which the displacement of structures was observed during muscle contraction. Differences in bladder base position between the two images were measured using vectors that could be determined on an ultrasound machine. The contraction time was 5 s each time, whereas the relaxation time was twice as long. The patient repeated this activity 10 times in each of the 10 series, with a rest time of 30 s between each series.

Training was performed by assessing the change in bladder base location with a normal pelvic floor muscle tone [35]. General Electric’s Voluson E10 ultrasound device was used for therapy. The therapies took place every day from Monday to Friday and lasted 2 weeks. All treatments were performed by one therapist.

Patients from group B underwent the electrostimulation of the pelvic floor muscles. The treatments were performed in the supine position on a couch with the lower limbs placed on a set of wedges. Electrostimulation was performed with the use of electrodes placed in the patients’ vagina. Treatments were performed using Kegel Plus Professional electrostimulation. Each of the 10 treatments lasted 30 min. The parameters for the procedure were consistent with those reported in the literature as being the most effective in the treatment of stress urinary incontinence [36].

After electrostimulation, the patients underwent a five-minute biofeedback training. The patients were asked to perform the contractions and relaxation of the pelvic floor muscles on their own when the game installed on the training device (MyoPlus 4 Pro Electromyograph) required from them. Using an endovaginal electrode receiving muscle signals, the pelvic floor muscle tension was imaged on the screen of the device in real time as a displayed electromyogram. During properly generated muscle tension, the indicator moved upward, whereas it did not change its position in the absence of tension, and when the strength of contraction decreased before the indicated time, it also decreased. The contraction time was 5 s. This was followed by a 10 s pause. The duration of a single training period was 5 min.

In patients from group C, no therapy was implemented; only the control measurements were the same as those in groups A and B.

The results of the study were collected in an Excel spreadsheet and then statistically analyzed using R Project [37]. The following was calculated as a part of our establishment of primary descriptive characteristics for measurable properties. Before starting the statistical analysis of the obtained results, the conformity of their distribution with the normal distribution was assessed using the Shapiro–Wilk test. Due to non-compliance using Kruskal–Wallis nonparametric analysis of variance, the significance level was *p* < 0.05.

## 3. Results

Overall, 75 women were included in the study. Ultimately, only 60 met all of the inclusion and exclusion criteria for the project. The characteristics of the examined group are presented in Table 1.

Inclusion and exclusion criteria are presented in Table 2.

**Figure 1:**
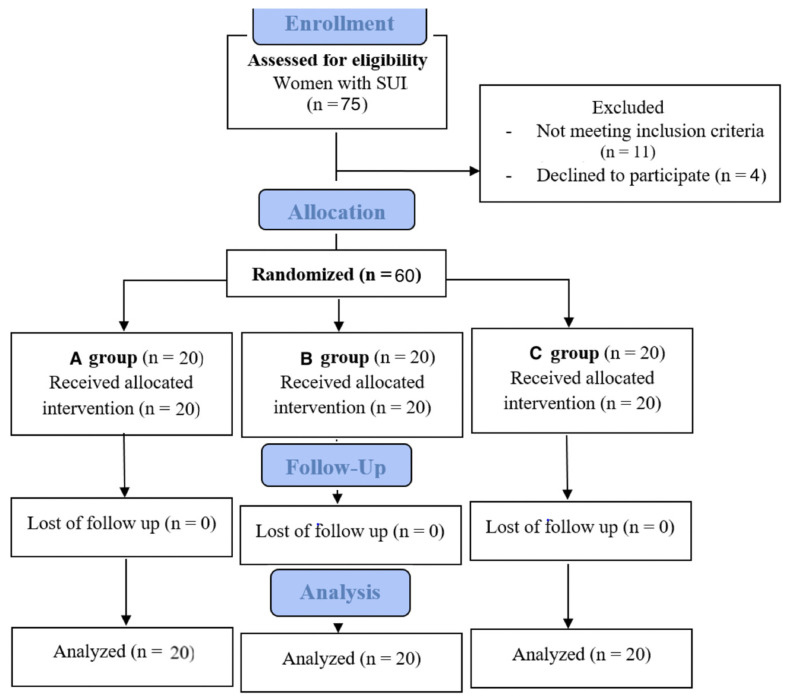
Flow chart of patients in study.

Figure 1 is a flow chart of the patients at each stage of the study. After the end of therapy, in both group A—subjected to sonofeedback training—and in the group B—in which pelvic floor muscle electrostimulation with biofeedback training was used—there was an upward trend observed in the bioelectrical activity of pelvic floor muscles evaluated during electromyography. Nevertheless, the results were not statistically significant, which may have been due to the small number of patients.

Table 3 shows that the results in all three groups before treatment, after 5 interventions, and at the end of treatment were not statistically significantly different. In the sonofeedback group, the final value increased by 1.1 µV compared to the initial value. After 10 interventions, the minimum value in this measurement increased by 0.40 µV, while the maximum value increased by 4.30 µV from the initial values. There were no statistically significant differences in the resting muscle bioelectrical activity in this group.

In the group that received pelvic floor muscle electrostimulation with biofeedback training (group B), the final value increased by 0.55 µV compared to the initial value. After a series of 10 interventions, the minimum value increased by 0.40 µV, while the maximum value decreased by 1.00 µV from the initial values. There were no statistically significant differences in resting muscle bioelectrical activity in this group.

Changes in pelvic floor muscle bioelectrical activity occurring in the control group (group C) showed that the final value decreased by 0.43 µV compared to the initial value. In the third measurement, the minimum value decreased by 0.20 µV while the maximum value decreased by 2.00 µV from the initial values. There were no statistically significant differences in resting muscle bioelectrical activity in this group.

Major highlights of the study are:

1. Sonofeedback seems to be comparably effective as the electrostimulation of pelvic floor muscle.
2. An upward trend in bioelectrical muscles activity, which was not statistically significant, was observed in both procedures.

## 4. Discussion

The effect of incontinence on quality of life depends largely on the severity of symptoms. In cases of significant discomfort, the performance of basic activities of daily living is impaired. In his study, Subak estimated that patients with symptoms of severe incontinence suffer from depressive symptoms in as many as 80% of cases. Among women with a low degree of incontinence, this number oscillates around 40% [38]. Irwin et al. have shown that patients with urinary incontinence very often have a sense of losing control over their bodies, which further increases their psychological discomfort and reduces their activities of daily living [39].

Importantly, even though the definition of stress urinary incontinence is simple—“urine loss during physical activity”—it frequently needs additional examinations to exclude other types of incontinence or its transitional causes. Diagnosis is made based on physical examination, urine analysis, ultrasound with the assessment of post-residual urine volume, and optionally drinking and voiding diary or questionnaires. Physical examination should contain vaginal examination and Bonney test [12].

According to the latest data, conservative treatment is effective in 80% of patients with I degree SUI and 50% of patients with II degree SUI. In patients with stage III, it helps to reduce the severity of symptoms and improves their quality of life. Properly performed PFM training is therefore extremely important for many women with SUI. Before starting it, patients should be instructed on how to perform it. The most common mistake is to tighten the abdominal muscles instead of PFMs. According to Moroni et al., training should consist of the maximal contraction of the PFMs for 5–10 s, and the daily number of repetitions can be as high as 300 [40].

The latest guidelines of the International Continence Society show that the electrostimulation of the PFMs aimed at reinforcing these muscles is an integral part of physiotherapy training [41]. In the available literature, there is wide variety in terms of the parameters of electrical current, electrode type, and their placement. The effectiveness of electrostimulation in the treatment of UI has been evaluated by many authors. Jha et al. [42] compared electrostimulation with traditional PFM training. This study was performed on 114 female patients with urinary incontinence. They found that both forms of physiotherapy had a beneficial effect on the urinary incontinence treatment, but did not conclude which was more effective [42]. On the other hand, Ma and Liu conducted a meta-analysis in which they compared the effectiveness of electrostimulation and electrostimulation combined with muscle training in patients with urinary incontinence. They showed that electrostimulation combined with muscle training is a more effective treatment method [43].

This form of therapy was also shown to be effective in our study. Although they do not show significant changes in the bioelectrical activity of the PFMs, the electrostimulation treatments with biofeedback training still demonstrate positive upward trend. This was confirmed by electromyographic findings. After treatment, the mean resting potential of the pelvic floor muscles increased by 0.55 µV from the initial value. However, the resulting changes were not statistically significant.

Ultrasonography is a single technique that has found application in the treatment of SUI. Thanks to ultrasonography, it has become possible to monitor muscle activity and thus improve muscle function. This therapy is referred to as sonofeedback. According to the recent literature, sonofeedback allows reinforcement of the PFMs [44]. Using this method seems to be an alternative to electrostimulation, especially since many women, despite its numerous advantages, resign from this treatment form. The most common cause is concern over the type of physical agent being used—electricity. Patients drop out of therapy equally often due to experiencing irritating and unpleasant sensations during the procedure.

Ultrasound imaging enables biofeedback assessment, as it provides real-time images of change in PFM tone. This allows it to be a tool for PFM re-education in people with incontinence problems. Sonofeedback is particularly beneficial in patients with uncoordinated contractions of the PFMs, displaying excessive tension and an impaired ability to initiate movement in a conscious manner. This information is particularly relevant in the context of the study by Liebergall-Wischnitzer et al. [45], who showed that exercise effectiveness decreases with age. After the age of 45, only 20% of patients perform them correctly. Biofeedback allows learning to consciously modify muscle tone by receiving auditory, visual, and sensory stimuli [45].

In their study, Eik-Nes et al. [46] enrolled 103 pregnant nulliparous women who were 20 weeks pregnant and had known stress urinary incontinence. They applied biofeedback training to all women. During therapy, they assessed pelvic floor muscle thickness gain and vaginal compression pressure. After the project, the researchers found 19 to 25 percent improvement in PFM function scores. They concluded that, as a source of biofeedback, ultrasound imaging provides information about the direction of PFM movement during contraction and functional tasks, and thus is an effective form of treatment. The researchers also emphasized that it is important to prevent the patient from exhibiting abdominal, thoracic, and lower extremity muscle tension during training [46]. Dietz et al. enrolled 212 women in their study, and found that 26% of the patients were unable to perform a proper PFM contraction, while up to 57% of them were able to perform the task after 5 min of biofeedback ultrasound training. In addition, they found that 62% of the studied women lowered their pelvic floor when attempting to perform a strong contraction of the PFMs, but performed the training correctly as early as after one biofeedback session. As with any type of biofeedback, the testing protocol must be carefully established [26].

It is worth noting that many factors may affect results achieved. One of them is position while performing pelvic floor muscles exercises: women with stress incontinence have lower muscle function compared to women without SUI in standing or sitting positions, but not in a lying position [47].

Our own studies also support the effectiveness of sonofeedback in postmenopausal women with SUI. Although the results did not show statistically significant changes in the bioelectrical activity of the PFMs, a positive upward trend can be noted, as confirmed by the results obtained from the electromyography test. After therapy, the mean resting potential increased by 1.1 µV from the initial value. In comparison, this change was 0.55 µV in the electrostimulation treatment group with biofeedback training.

It is necessary to search for effective treatment methods, as the number of women with the problem of incontinence is predicted to increase every year. The results obtained in this study indicate that sonofeedback may be one of them. This method increased the bioelectrical activity of the PFMs. The correct bioelectrical activity of the PFMs is extremely important in the activity of these muscles. The reduced resting potential of the PFMs is associated with the presence of SUI. A weakened myofascial and fascial–ligamentous system corresponds with developing symptoms.

As significantly lower measurements in electromyography are attained in individuals with SUI, bioelectrical activity value can be an important tool to confirm its absence.

In a Polish study, a diagnostic cutoff for differentiating women with SUI symptoms and healthy individuals was established: “baseline”—3.7 µV, contraction—11.33 µV and “rest”—3.89 µV [48].

Physiotherapeutic methods contributing to increasing the bioelectrical activity of muscles, such as sonofeedback, will therefore have a direct effect on reducing the severity of SUI symptoms, which is why it is so important to find such methods and apply them in everyday clinical practice.

A major limitation of our preliminary study is the relatively small number of women included, a lack of power analysis for the study, and the lack of long-term follow-up verifying the durability of the effect obtained. All the weaknesses could be compensated for in a prospective study.

The major strengths of the study are its feasibility and the possibility of its clinical appliance in patients with contraindications for electrostimulation. Moreover, the control group and the electrostimulation and sonofeedback group in the study may have additional impact on conclusions drawn.

Future studies with larger numbers of patients are recommended, as it is likely that too few participants may have been the reason for the lack of statistically significant changes in the parameters assessed. In addition, it is also advisable to conduct studies on patients with other medical conditions, especially those in which electrostimulation is contraindicated. Oncological patients with increased muscle tone can be such a group. There are no scientific reports that would evaluate the efficacy of sonofeedback in this patient group, and using this method seems reasonable, as it is completely noninvasive and safe.

## 5. Conclusions

Both sonofeedback and the transvaginal electrostimulation of the PFMs did not increase the bioelectrical activity of these muscles. Nevertheless, there was an upward trend observed.

Based on the results obtained, it can be concluded that the sonofeedback method is comparably effective in stimulating the bioelectrical activity of the pelvic floor muscles to the standard electrostimulation method with biofeedback training.

It is necessary to conduct further studies to assess the effectiveness of the sonofeedback method, especially in clinical application and with long-term observation.

## Figures and Tables

**Table 1:** Characteristics of patients.

Number of People		*n* = 60
Age [years]	Min–max	45–65
	Mean	57
	Standard deviation	6.26
	Median	57
Body height [m]	Min–max	1.49–1.72
	Mean	1.61
	Standard deviation	0.05
	Median	1.62
Body weight [kg]	Min–max	48–100
	Mean	69.94
	Standard deviation	11.65
	Median	69.5
BMI [kg/m^2^]	Min–max	17.63–38.86
	Mean	27.07
	Standard deviation	4.69
	Median	26.74
Number of births	Min–max	1–4
	Mean	2.10
	Standard deviation	0.71
	Median	2
Type of delivery	birth by the forces of nature	55 (91.67%)
	cesarean section	5 (8.33%)
Comorbidities	Yes	49 (81.67%)
	No	11(18.33%)
The circumstances of the appearance of the first symptoms of SUI	after delivery	3 (5%)
	after gynecological surgery	22 (36.67%)
	after menopause	26 (43.33%)
	the patient is unable to give the circumstances	9 (15%)

**Table 2:** Study inclusion and exclusion criteria.

Inclusion Criteria	Exclusion Criteria
– Grade II SUI confirmed by a gynecologist; – Age 45–65; – Postmenopausal age; – No health contraindications to the use of therapy; – SUI occurrence for more than 5 years; – Obtaining informed written consent from the patient.	– An implanted pacemaker; – Lower urinary tract infections; – Decreased nerve excitability; – Difficulty cooperating with the studied person; – Organ prolapse; – Previous pelvic surgery; – SUI pharmacological treatments; – Other conservative treatments for SUI.

**Table 3:** Intergroup comparisons of resting PFM bioelectrical activity measured by EMG before, after 5 treatments, and after therapy, and also the *p*-value between groups also at different times.

	EMG [µV]			*p*-Value
	Before Therapy	After 5 Therapies	After Therapy	Kruskal–Wallis Test
SONOFEEDBACK (A)				
n	20	20	20	
median	3.70	4.05	4.45	
minimum	1.20	1.70	1.60	0.8584
maximum	6.10	9.40	10.40	
v	39	45	46	
ELECTROSTIMULATION (B)				
n	20	20	20	
median	3.65	3.75	4.00	
minimum	1.20	1.20	1.60	0.8617
maximum	7.90	7.50	6.90	
v	46	45	38	
CONTROL (C)				
n	20	20	20	
median	3.95	4.00	3.75	
minimum	1.40	1.70	1.20	0.9899
maximum	10.70	12.20	8.70	
v	57	61	46	
*p*-value				
Kruskal–Wallis test	0.7563	0.8879	0.9410	

n = number of observations, v = coefficient of variation.

## Data Availability

The datasets used and/or analyzed during the current study are available from the corresponding author on reasonable request.
